# Influence of TIMP-1/MMP-9 ratio on the severity and mortality in sepsis

**DOI:** 10.1186/cc9697

**Published:** 2011-03-11

**Authors:** L Lorente, MM Martín, J Solé-Violán, J Blanquer, L Labarta, C Díaz, JM Borreguero-León, JA Páramo

**Affiliations:** 1 Hospital Universitario de Canarias, La Laguna, Spain; 2 Hospital Universitario Nuestra Señora de Candelaria, Santa Cruz de Tenerife, Spain; 3 Hospital Universitario Dr Negrín, Las Palmas de Gran Canaria, Spain; 4 Hospital Clínico Universitario de Valencia, Spain; 5 Hospital San Jorge, Huesca, Spain; 6 Hospital Insular, Las Palmas de Gran Canaria, Spain; 7 CIMA-Universidad de Navarra, Pamplona, Spain

## Introduction

The role of matrix metalloproteinases (MMPs) and tissue inhibitors of matrix metalloproteinases (TIMPs) in sepsis remains unclear. MMPs play a role facilitating the recruitment of leucocytes from the bloodstream (by proteolysis of the basement membrane) and modulating inflammatory response [1]. Besides, there has been reported a positive association between circulating levels of TIMP-1 and plasminogen activator inhibitor (PAI)-1 in healthy adults [2] and myocardial infarction [3]. In addition there are *in vitro *studies showing that MMP-9 inhibits platelet aggregation [4,5]. Thus a high TIMP-1/MMP-9 ratio could contribute to a prothrombotic state, and the development of organ dysfunction and finally death in septic patients. The objectives of this study were to investigate the time course of MMP-9, MMP-10 and TIMP-1 levels, and the association with sepsis severity and PAI-1 levels.

## Methods

This was a multicenter, observational and prospective study carried out in six Spanish ICUs. We included 192 (125 surviving and 67 nonsurviving) patients with severe sepsis. We obtained blood samples at three moments (time of diagnosis, 72 hours and 7 days) for the determination of MMP-9, TIMP-1, TNFα, IL-10 and PAI-1 levels. We assessed survival at 30 days as the endpoint.

## Results

Nonsurvivor patients showed at the three moments lower MMP-9 levels, higher TIMP-1 levels and higher TIMP-1/MMP-9 ratios than survivors. There were at the three moments an association of the TIMP-1/MMP-9 ratio with lactic acid levels, SOFA score, PAI-1 levels, TNFα and IL-10. Logistic regression analysis showed that TIMP-1 levels, lactic acid levels and SOFA score were associated with death at 30 days.

## Conclusions

To our knowledge, this study includes the largest series reporting data on MMP levels in sepsis. The novel findings of our study are that nonsurviving septic patients showed a persistent higher TIMP-1/MMP-9 ratio during the first week than survivors. From a therapeutic perspective, the development of modulators of MMP/TIMP activity could be used as a new class of drugs for the treatment of severe sepsis.
